# Development of community strategies supporting brief alcohol advice in three Latin American countries: a protocol

**DOI:** 10.1093/heapro/daab192

**Published:** 2021-11-29

**Authors:** Adriana Solovei, Liesbeth Mercken, Eva Jané-Llopis, Inés Bustamante, Silvia Evers, Antoni Gual, Perla Medina, Juliana Mejía-Trujillo, Guillermina Natera-Rey, Amy O’Donnell, Augusto Pérez-Gómez, Marina Piazza, Hein de Vries, Peter Anderson

**Affiliations:** 1 Department of Health Promotion, CAPHRI Care and Public Health Research Institute, Maastricht University, Maastricht, The Netherlands; 2 Univ. Ramon Llull, ESADE, Barcelona, Spain; 3 Institute for Mental Health Policy Research, CAMH, Toronto, Canada; 4 School of Public Health and Administration, Universidad Peruana Cayetano Heredia, Lima, Peru; 5 Department of Health Services Research, CAPHRI Care and Public Health Research Institute, Maastricht University, Maastricht, The Netherlands; 6 Addictions Unit. Psychiatry Dept. Hospital Clínic, Barcelona, Spain; 7 Red de Trastornos Adictivos. Instituto Carlos III, Madrid, Spain; 8 Institut d’Investigacions Biomèdiques August Pi Sunyer (IDIBAPS), Barcelona, Spain; 9 Instituto Nacional de Psiquiatría Ramón de la Fuente Muñiz, Ciudad de México, Mexico; 10 Corporación Nuevos Rumbos1, Bogotá, Colombia; 11 Population Health Sciences Institute, Newcastle University, Newcastle upon Tyne, UK

**Keywords:** brief alcohol advice, community actions, primary healthcare, implementation, Latin America

## Abstract

Brief alcohol advice offered to patients was shown to be a clinically- and cost-effective intervention to prevent and manage alcohol-related health harm. However, this intervention is not yet optimally implemented in practice. A suggested strategy to improve the implementation of brief alcohol advice is through community actions which would enhance the environment in which primary healthcare providers must deliver the intervention. However, there has been scarce research conducted to date regarding which community actions have most influence on the adoption and implementation of brief alcohol advice. The current protocol presents the development of a package of community actions to be implemented in three Latin American municipalities, in Colombia, Mexico and Peru. The community actions were based on the Institute for Health Care Improvement’s framework for going to full scale, and include: (i) involvement of a Community Advisory Board, (ii) involvement of a project champion, (iii) adoption mechanisms, (iv) support systems and (v) a communication campaign. By presenting a protocol for developing community actions with input from local stakeholders, this article contributes to advancing the public health field of alcohol prevention by potentially stimulating the sustainable adoption and implementation of brief alcohol advice in routine practice.

## INTRODUCTION

Alcohol is the ninth leading risk factor for morbidity and premature death, responsible for over 5% of mortality worldwide, and more than 5% of the global burden of disease and injury, including non-communicable diseases (e.g. liver cirrhosis, cardiovascular diseases, cancers), neuropsychiatric conditions and injuries from violence and road-traffic crashes (Rehm *et al.*, 2009; WHO, 2019). In the three countries addressed in this protocol, alcohol ranks even higher, representing the fourth leading risk factor in Colombia and Peru, and the fifth in Mexico (GBD 2015 Risk Factors Collaborators, 2016). In spite of this evidence, about 80% of heavy drinkers worldwide fail to receive appropriate advice or treatment (WHO, 2008), which highlights the importance of implementing interventions that can increase patients’ access to alcohol advice and/or treatment. The purpose of this article is to present the development of a package of community actions (to be implemented in the three above-mentioned Latin American countries), aiming to support primary healthcare (PHC) providers to adopt and deliver brief alcohol advice to their patients (as explained in more detail below).

PHC is a recommended setting for addressing harmful alcohol drinking, because of the high frequency of encounters between patients and PHC providers and its extensive coverage of the general population (Anderson, 1996). Specifically, assessing a patient’s alcohol consumption using a short validated instrument such as the Alcohol Use Disorder Identification Test-Consumption version (Bush *et al.*, 1998), and providing brief advice to those identified as heavy drinkers, has been shown to be clinically- (O’Donnell *et al.*, 2014; Platt *et al.*, 2016; Kaner *et al.*, 2018) and cost-effective (Solberg *et al.*, 2008; Anderson *et al.*, 2009) in the prevention and management of alcohol-related harm. Despite such evidence, however, these interventions are not yet optimally implemented in practice (Johnson *et al.*, 2011; Wilson *et al.*, 2011; Abidi *et al.*, 2016). Some of the most notable barriers to routine delivery of alcohol advice include: the (perceived) lack of time of PHC providers; the lack of training, resources and/or supportive policy environment; and concerns amongst a substantial number of PHC providers that patients will be offended if the subject of alcohol is brought up, and will not listen to the offered advice (Nilsen, 2010; O’Donnell *et al.*, 2014).

A comprehensive multi-country World Health Organisation (WHO) study of the implementation of brief alcohol advice in PHC suggested that in order to overcome these barriers, it is important to both reframe views of individual healthcare professionals and patients about alcohol and to embed delivery of brief alcohol advice into a set of wider community actions which would enhance the environment in which PHC providers must deliver the intervention (WHO, 2006). The conclusions of the WHO study build on a long line of work, dating back to the Maudsley Alcohol Pilot Project that was set up in the United Kingdom in the 1970s to make practical recommendations for an improved local response to dealing with drinking problems (Shaw *et al.*, 1978). The project, which subsequently informed the United Kingdom’s Royal College of General Practitioners’ report on alcohol (Anderson and Clement, 1987), was premised on the view that to respond to drinking problems adequately, PHC providers need to be involved, trained and supported at the community level. Several studies showed empirically that community-based interventions are indeed highly relevant in the context of alcohol prevention, by shifting positive attitudes towards desired alcohol-related behaviours (Casswell and Gilmore, 1989), ensuring overall acceptability of such interventions (Czech *et al.*, 2010) and being potentially cost effective (Holder, 2000).

### Community stakeholders

Shakeshaft *et al.* (Shakeshaft et al., 2014) argue for the importance of including key community stakeholders (e.g. representatives of the municipal administration) in community-based interventions in the context of alcohol prevention, as this is expected to increase the visibility and acceptability of the intervention. A way of involving such actors in community-based interventions is through Community Advisory Boards (CABs) (Strauss *et al.*, 2001; Newman *et al.*, 2011; Rahman *et al.*, 2019). A CAB is a group formed of relevant stakeholders (e.g. delegates of a regional health department, healthcare professionals, patient representatives, civil society members) who offer regular advice, feedback and suggestions, and generally represent the views and needs of the community in the implementation of a health programme. In the field of alcohol prevention, CABs may ensure that research methods are culturally appropriate and applicable to the local contexts (Morojele *et al.*, 2006), and may identify and signal potential barriers and facilitators in the implementation of the alcohol-related intervention (Dickerson *et al.*, 2016).

Moreover, the efforts of CABs in the development and maintenance of supportive actions are often strengthened by a project champion, a person who advocates the implementation of a new (health) programme and generates support for its adoption (Markham and Griffin, 1998; Vendetti *et al.*, 2017). In the context of brief alcohol advice, several studies have shown that the involvement of a project champion is an important success factor in the implementation of the programme (Gifford *et al.*, 2012; Anderson *et al.*, 2017c), for example by promoting the adoption of the intervention to PHC providers, by ensuring regular communication with the PHC managers and providers in order to foresee and overcome barriers, and by promoting the sustainability and scalability of the intervention.

### Community actions

An evidence-based framework that can guide the development of community actions with input from key stakeholders is the Institute for Health Care Improvement’s (IHI) framework for going to full scale, developed by Barker *et al.* (Barker *et al.*, 2015). This framework illustrates several important types of community actions aimed at enhancing the adoption and scalability of a health intervention, which are briefly outlined below. In the set-up phase of an intervention, they emphasize the importance of implementing a range of adoption mechanisms, that is, carrying out a set of actions that would foster the acceptance and initial implementation of an intervention by the relevant stakeholders. Such adoption mechanisms may include highlighting the simplicity and/or benefits of the innovation to the relevant stakeholders or identifying potential organizational barriers along with solutions to overcome them (Rogers, 2010). Implementing adoption mechanisms is important in the context of alcohol prevention because the topic of alcohol is often linked to stigmatization (Room, 2005), is perceived as a taboo subject to discuss in a medical setting (Mules *et al.*, 2012) and is associated with limited health literacy about its harms (Rundle-Thiele *et al.*, 2017). These can be serious impediments to the adoption of interventions by healthcare providers. The implementation of adoption mechanisms may help healthcare providers to overcome these barriers, thereby stimulating them to deliver preventive interventions to their patients.

Further, Barker *et al.* (Barker *et al.*, 2015) highlight that throughout the lifecycle of the intervention, various support systems should be implemented. Support systems refer to actions that provide an encouraging environment for delivering the intervention and diminish the implementation barriers. Such actions may include regular performance feedback given to the stakeholders involved or actively offering solutions to encountered barriers throughout the implementation process. Support systems are important in the context of alcohol prevention interventions, especially when such interventions require a continuous involvement of the implementers, for example PHC providers. For example, in the context of brief alcohol advice, a common barrier mentioned by PHC providers is the perceived lack of time and managerial support in addressing the topic of alcohol with their patients (Roche and Freeman, 2004; Nilsen *et al.*, 2006), which results in decreased implementation. In such cases, implementers can be helped to overcome the experienced barriers through regularly implemented support systems, as those mentioned above.

Moreover, communication campaigns are often part of community actions aimed at supporting alcohol prevention interventions (Dejong and Atkin, 1995; Elder *et al.*, 2004; Young *et al.*, 2018). Communication campaigns are organized communication activities, directed at a particular population for a period of time, to achieve a predetermined goal, for example convincing relevant stakeholders to participate in an intervention (Snyder, 2007; Rogers, 2010). General advantages of communication campaigns in health promotion activities include the ability to reach large (or targeted) audiences at a relatively low cost per capita (Wakefield *et al.*, 2010). Communication campaigns fit well in the scope of alcohol prevention efforts, for example by promoting awareness at the population level regarding risks of alcohol abuse (Shakeshaft *et al.*, 2014) or encouraging interpersonal communication on the topic of alcohol (Hendriks *et al.*, 2014).

To our knowledge, there has been limited research conducted to date regarding which community actions may impact PHC providers’ adoption and implementation of brief alcohol advice. Moreover, no such study has been previously carried out in Latin America. To fill this literature gap, this article aims to describe the protocol for the development of a package of community actions to support the implementation of PHC based alcohol interventions in three upper-middle-income countries in Latin America, namely Colombia, Mexico and Peru. This article is part of the larger SCALA study that aims to scale-up PHC-based prevention and management of alcohol use disorder and depression at the municipal level in the three countries (Jané-Llopis *et al.*, 2020).

## METHODS AND OUTCOMES

### SCALA study design and sample

SCALA is a quasi-experimental study rooted in implementation science, which seeks to increase the delivery of evidence-based approaches to the prevention and management of alcohol use disorder and comorbid depression in Colombia, Mexico and Peru. SCALA will use a multi-component approach to boost implementation, which comprises: locally tailored care pathway, clinical intervention materials (two versions: either standard or more intensive); training sessions (two versions: either standard or more intensive) with PHC providers; and introduction of community actions. The larger SCALA study consists of four arms (arm 1: care as usual; arm 2: implements standard training and clinical package; arm 3: implements standard training and clinical package, *and* community actions; arm 4: implements more intensive training and clinical package, *and* community actions). Implementation will last 18 months. By month 6 of implementation, the non-superiority of arm 4 over arm 3 will be tested. If the difference of the cumulative coverage of patients whose alcohol consumption is measured is less than 10% between the two arms, arm 4 will be replaced by arm 3.

### Participants and power calculations

In each country, there is one intervention municipality and one comparator municipality, each including 9–10 participating PHC centres (PHCCs). Municipal areas are investigator-selected, ensuring comparability among the intervention and control municipal areas in terms of size and socioeconomic characteristics. Sufficient geographical separation between the municipal areas in each country was taken into account, to minimize potential contamination effects of the control municipality from the intervention municipality. PHCCs were invited to join the study, through face-to-face meetings and/or telephone calls, until a minimum of nine PHCCs per municipal area (intervention and control) within each of the three countries were achieved. In the end, 58 PHCCs were recruited, 29 in the intervention municipal areas and 29 in the control municipal areas. All PHCCs are part of the public healthcare systems in each of the three countries, under the jurisdiction of regional health departments (in all three countries, the national healthcare systems are comparable, consisting of both public and private health services). Within the control municipal area, 14 PHCCs were randomly allocated to arm 1, and 15 PHCCs—to arm 2. Within the intervention municipal area, 15 PHCCs were randomly allocated to arm 3, and 14 PHCCs—to arm 4. Random allocation was stratified by country and undertaken using Excel random number generator. In total, approximately 600 PHC providers agreed to participate in the study, by signing the informed consent. The number of participating PHCCs (which ultimately determines the sample size of participating PHC providers, who register voluntarily) has been defined based on the primary outcomes in the whole SCALA study (i.e. comparing the proportions of patients who receive brief advice among the study arms). The power calculations are based on the assumptions that the average size of a PHCC included in the study is 15 000 adults and the average of consultations is 1500 per month. Based on results from a comparable study in a European setting (Anderson *et al.*, 2017b), it is expected that after 12 months of implementation, 3.25% of the registered adult population to have had their alcohol consumption measured in the control condition (arm 1), and 7.5% (arm 2). For arms 3 and 4, no precise empirical data are available to allow estimations, however, at least 15% of the registered adult population would need to have their alcohol consumption measured in these arms in order to consider the effect of community actions worthwhile. For comparing differences between arms, for a power of at least 80% at a significance level of 5% (Donner and Klar, 2010), 12–15 PHCCs per arm are needed (i.e. 4-5 PHCCs per arm in each country).

### Development of SCALA community actions

**Fig. 1:**
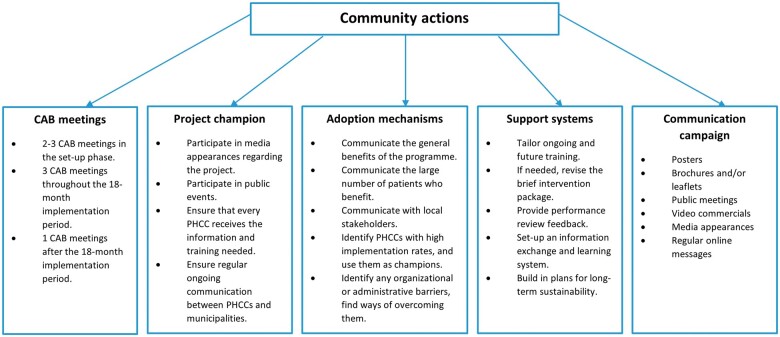
Overview of the SCALA community actions.

Community actions, as provided in study arms 3 and 4, are operationalized through a package of activities that will be implemented in the three intervention municipalities in Peru (Callao), Colombia (Soacha) and Mexico (parts of Mexico City). These actions have been developed based on the IHI framework for going to full scale (Barker *et al.*, 2015), with input from and in collaboration with local stakeholders and the public health experts involved in the project. In the preparation phase, the local research teams in the three countries exchanged ideas and best practices, leading to the development of three comparable community actions plans. The aim of the community actions has been defined as to increase the support perceived by PHC providers to deliver brief alcohol advice to their patients. In each of the three countries, the planned community actions consist of five blocks (as depicted in Figure 1): (i) involvement of the CAB; (ii) involvement of one or more project champions; (iii) implementation of adoption mechanisms; (iv) implementation of support systems and (v) implementation of a communication campaign. Below, each of these activities is explained.

#### Community advisory board

In each intervention municipality, a CAB was formed in the first months of the project, specifically from April to May 2018. The three CABs have comparable compositions, each consisting of 10–12 members representing relevant organizations and/or sectors such as regional health departments, academia, mass media and non-profit organizations. The core aims of the CABs have been defined as (i) to provide input and feedback into the development of the community actions, (ii) to be involved (when necessary) in the implementation of the community actions, (iii) to provide suggestions regarding the clinical and training package, and its delivery in PHC, and (iv) to provide suggestions with regard to any other issues (e.g. organizational barriers) that may arise throughout the implementation period. In line with these aims, before the start of the implementation period, two to three CAB meetings were held in each intervention municipality, to discuss the set-up of the project, the materials used in the intervention, and the planned community actions. Throughout the 18-month implementation period, CAB meetings will take place approximately once in 6 months (or more often on an ‘as needed’ basis). In addition, depending on their availability and areas of expertise/experience, it has been agreed that individual CAB members will give advice and feedback during face-to-face meetings, phone calls and online communication with the local research teams. CAB members will also be directly involved in the implementation of some community actions (e.g. media appearances, participation in public events), as specified below.

In the set-up phase of the project (i.e. before the start of the implementation period), although the three CABs were not directly involved in the selection and recruitment of PHCCs, their support and endorsement towards the project contributed to the PHCCs’ managers and PHC providers’ openness to participate in the study. Moreover, the CABs provided input towards ensuring that PHC provider priorities will be taken into account in the implementation of the programme. For example, in all three countries, CAB members suggested simplifying the brief alcohol advice pathway in order to deal with providers’ (perceived) lack of time to deliver the intervention.

The CAB members also provided feedback on the tailoring of the clinical package materials (e.g. leaflets for patients, guidance materials for the PHC providers) and on the training delivered in the programme, in order to ensure an optimal harmonization with municipal and country realities. Clinical package materials and training programmes were initially developed by research partners in Europe, based on existing formats used in UK and Spain, respectively, and were afterwards translated, tailored and adapted to each of the three Latin American countries. The final versions of the materials and training structure can be accessed on the project website (scalaproject.eu), with the content of the materials and training being similar in the three countries. Tailoring suggestions from the CAB members referred mostly to concretizing the definitions of standard drinks in the clinical package materials, by including examples of drinks specific to each country (e.g. aguardiente in Colombia, tequilla Mexico, pulque in Peru). Moreover, CAB members made suggestions regarding the key societal problems caused by alcohol in the respective country to be mentioned in the materials, taking into account, among others, gender-related differences and priorities. For example, in Peru, CAB members suggested highlighting the issue of violence against women as one of the core problems caused by alcohol. In Mexico, it was suggested to include examples of alcohol harms specific for men, for example, erectile dysfunctions, and for women, for example, disturbances of the menstrual cycle. In Colombia, it was recommended to emphasize more clearly that alcohol use raises the risk of getting various types of cancers and mental health problems, such as depression and anxiety.

In all three countries, the CABs also gave input regarding institutions to be suggested for help-seeking patients, relevant for each country, and which included: telephone numbers, social media platforms (e.g. Facebook, Whatsapp) and websites. In Colombia, it was additionally suggested to clarify whether the recommended services are free of cost. Moreover, in all three countries, CABs’ suggestions regarding adapting the training were, for example, to emphasize strategies for providers in dealing with reluctant patients during a brief alcohol advice session. More specifically, in Colombia, it was suggested to develop video tutorials, in addition to the training, that can be easily accessed by the participating providers whenever they need additional clarifications about the intervention delivery. In Mexico, on the other hand, it was recommended to develop additional printed instructions for providers, for example, a quick guide of the intervention that can be placed on providers’ desks. In Peru, it was suggested to add more focus in the training on psychologists and other mental health workers (in addition to the medical healthcare providers), thereby reflecting the key role of mental health workers in the alcohol prevention domain in the country.

#### Project champion

One or two project champions were selected in each intervention municipality at the outset of the study. They are CAB members and professionals in (public) health institutions of the intervention municipalities (e.g. representative of the municipal hospital, health advocacy expert). Throughout the implementation period, the project champions will be invited to take on roles such as: (i) facilitating agreement within the municipality and health systems on shared goals and metrics; (ii) assessing and acting on relevant community resources; (iii) working at the systems level to make relevant practice changes for sustainability; (iv) supporting PHCCs to access, and manage needed services; (v) ensuring regular ongoing communication between PHCCs and municipalities. This will be achieved through regular meetings with the PHCCs throughout the implementation period, regular media appearances and participation in public events on project-related topics. Moreover, the project champion will offer suggestions for ensuring the sustainability of the intervention at municipal and/or national level.

#### Adoption mechanisms

Five adoption mechanisms have been included in the package of community actions and will be implemented in each of the three intervention municipalities. First, during the recruitment period and in the first months of implementation, formal and informal meetings will be organized where the local research team and the project champion(s) will communicate to PHC providers and representatives of the PHCCs the simplicity of the programme and its benefits to patients. The reasoning for this adoption mechanism is that a simple health programme is more likely to be adopted than a complex one (Greenhalgh *et al.*, 2005). Second, during formal and informal meetings, in the recruitment period and the first months of implementation, the local research team and the project champion will communicate to PHC providers the large gap between the number of patients who need advice regarding their alcohol use and the number of patients who actually receive it. The potential of the SCALA programme to fill this gap will be emphasized, as it is essential to highlight the advantages of a health programme to increase its success of adoption (Rogers, 2010).

Third, during CAB meetings and regular communication, the local research teams will emphasize to relevant stakeholders (e.g. CAB members, PHCC administration) their important role in promoting brief alcohol advice programmes. The involvement of local stakeholders is important because receiving information from various credible stakeholders regarding an innovation also increases its adoption rate (Brinol and Petty, 2009) through the ‘multiple source effect’ (Harkins and Petty, 1987). Fourth, PHC providers who have high advice-giving rates will be identified. These PHC providers will be champions who communicate to other providers and communities that ‘it can be done’. Successful implementation examples are another important facilitator of the intervention adoption process (Barker *et al.*, 2015). To ensure this, during the baseline measurement period and throughout the implementation period, those PHCCs and PHC providers who advise high proportions of patients will be identified. Subsequently, they will be invited to share their experiences and provide positive messages regarding brief alcohol advice, through regular internal communication, as well as through media appearances. Fifth, organizational issues or administrative policies that act as barriers will be timely identified, along with ways of overcoming them through discussions with PHC providers on an ongoing basis. Barriers to adopt and implement brief alcohol advice can vary among countries and organizations (Johnson *et al.*, 2011). To ensure accurate identification of such barriers, open questions will be regularly asked of healthcare providers and CABs’ members during formal and informal meetings.

#### Support systems

Five support systems have been included in the package of community actions and will be implemented in each intervention municipality. First, training packages will be tailored to the local PHC providers’ needs, as evidence shows that effective training in the delivery of brief alcohol advice should cover the actual needs of the PHC providers (Seale *et al.*, 2005). Tailoring of training will be based on regular input received from PHC providers and PHCC managers, through informal meetings conducted in the first months of implementation. Second, revisions of the brief advice package will be done, if needed, to ensure that the materials are in line with the needs of the PHC providers and patients. Again, during the first months of the implementation, there will be regular communication with PHC providers to identify whether the intervention packages and the care pathway require any modification.

Third, performance review feedback will be provided to the PHC providers. Feedback is an effective mechanism to promote active and continuous participation in an intervention (Barker *et al.*, 2015). During the 18-month SCALA implementation period, there will be regular feedback given to the PHCCs in the three intervention municipalities on their performance of alcohol advice giving. Specifically, monthly data on advice-giving rates delivered at each PHCC will be provided to the PHCC managers in either written or face-to-face communication format, by the local research teams. Moreover, positive feedback will be given to high-performing PHCCs and healthcare providers to encourage and maintain this. PHCCs and PHC providers with lower advice-giving rates will be asked about any improvements and changes that could be introduced to help improve performance.

Fourth, an information exchange and learning system for participating PHC providers will be set-up. Interactive learning platforms allow the exchange of ideas and experiences among adopters and can increase knowledge, skills and motivation to participate in the intervention (Schouten *et al.*, 2008; McCannon and Perla, 2009). During the SCALA implementation period, a digital learning system will be created, where for example, online project progress messages will be regularly circulated, and through which PHC providers and local stakeholders will be able to exchange ideas and learning to support improved delivery of alcohol advice.

Fifth, plans for long-term sustainability of the SCALA programme will be built-in from the outset, aiming to integrate the intervention in the health system. Timely planning for a health programme’s sustainability is crucial for ensuring that the programme has the capability to be maintained after the end of a research project (Gruen *et al.*, 2008). In line with this, the programme’s sustainability will be an ongoing agenda item of the CAB meetings, and local research partners will receive input directly from PHC providers and PHCC managers who implement the SCALA protocol (e.g. measuring the necessary costs to set-up and implement the intervention, or the possibility to integrate the intervention in the existing public policy plans).

#### Communication campaign

In each of the three intervention municipalities, a communication campaign will be implemented, aiming to additionally increase the community support received and perceived by PHC providers in the adoption and maintenance phase of the health programme. The main activities of the communication campaign are specified below and are typically used in health communication campaigns (Snyder, 2007). The activities will be developed locally using input from the CABs and other members of the communities. In all three countries, it has been decided to implement a similar set of six communication activities, following collaboration and exchange of ideas and resources between the three local research teams. Where needed, the content of the communication materials will be adapted to the context of each country, by pilot testing them with members of the CAB, as well as in user panels with providers and/or patients. The six communication campaign activities are as follows (country differences in terms of planned implementation are minimal and specified below).

First, posters will be placed in various public spaces promoting PHC-based alcohol advice, addressing the topic of alcohol-related problems and the benefits of discussing this during a PHCC visit. Posters will be strategically placed in areas where their target audience (i.e. PHCC patients, PHC providers) is most likely to frequent, such as the participating PHCCs and at other popular local venues, for example, pharmacies, bus stops and cafeterias. Second, leaflets and/or brochures about the health risks associated with alcohol, and the benefits of brief alcohol advice will be placed in PHCCs waiting halls. To promote optimum patient reach, leaflets will remain available in the campaign for the entire implementation period (i.e. until month 18). Third, short promotional videos about the benefits of brief alcohol advice will be displayed strategically on screens in the waiting rooms of PHCCs. Fourth, public events about the benefits of brief alcohol advice will be organized. Such public events, in the form of workshops, movie forums, presentations and public discussion in informal settings (e.g. civic centres, food markets, health fairs, libraries, cafes and bars) will allow reaching additional segments of the target audience (e.g. PHC providers and/or patients). These will be organized according to the available resources, at least once throughout the implementation period, in each of the three intervention municipalities. Fifth, throughout the implementation period, regular short messages on project-related topics will be sent to participating PHC providers in the three intervention municipalities. Topics of such messages may include relevant research on brief alcohol advice, health problems related to alcohol consumption, suggestions for conversations with patients about alcohol, SCALA success stories and SCALA public events. The messages will be delivered on channels such as online texting platforms (e.g. *Whatsapp*). Sixth, local media (TV/radio/print) appearances covering the subjects related to the project will be organized as frequently as possible throughout the 18-month implementation period. The planned execution and implementation of these communication campaign activities are similar in the three countries. A noteworthy difference is in Peru where the communication campaign activities will focus (next to the general population of PHC patients) on three specific target populations affected by alcohol (as suggested by the CAB members), namely: (i) persons in treatment of tuberculosis, (ii) persons at risk of sexually transmitted diseases and (iii) persons in violent families.

## DISCUSSION

This article presents the development protocol of a set of community actions to support PHC providers to deliver brief alcohol advice to their patients. The community actions have been designed using input from various local stakeholders, drawing on IHI Framework for going to full scale (Barker *et al.*, 2015) and include five key activities: (i) involvement of a CAB, (ii) involvement of a project champion, (iii) adoption mechanisms, (iv) support systems and (v) a communication campaign.

A strength of the protocol is that it has been developed and will be implemented in ‘real-life’ municipal settings, thus increasing its external validity and enhancing the potential to apply it to other settings and populations (Bryman, 2016). This offers a basis for further exploration of whether community actions are successful in promoting the delivery of brief alcohol advice in PHC. Moreover, the planned implementation period duration of 18 months, which is longer than in typical brief advice implementation studies (Funk *et al.*, 2005; Anderson, *et al.*, 2017a) will allow detecting long-term effects of the community actions and whether these differ between participating countries. Another strength of the study lies in its focus on implementation research. There is a substantial body of evidence showing that brief alcohol advice in a PHC setting is an effective measure for decreasing alcohol consumption among large segments of the population (Kaner *et al.*, 2018). However, more studies are needed to understand which mechanisms are most effective to increase the likelihood of the adoption and implementation of brief alcohol advice (Nilsen, 2010). The methodology presented in this article for developing a package of community actions with the involvement of local stakeholders can contribute to advancing the alcohol prevention field and, therefore, prove relevant for public health researchers and practitioners implementing similar interventions in Latin America, as well as in other regions worldwide, given adequate tailoring and adaptation. The presented package of planned community actions has the potential to stimulate the sustainable implementation of a health intervention proven to be effective at reducing alcohol-related harm. Future implementation science studies can benefit from investigating the effectiveness, as well as the implementation process (including barriers and facilitators) of the presented planned community actions, aiding an optimal implementation in different contexts.

It is also important to acknowledge some key limitations. Although the development of the package of community actions was done based on input provided by local CABs, the project’s design and resources did not allow for a systematic needs assessment from the PHC provider and patient perspective regarding the supportive actions. As a result, some community actions may need to be changed or adapted during the implementation period. Any such changes and/or adaptations to the community actions plans will be monitored and documented throughout the 18-month implementation period. Another limitation is that, in the SCALA study, PHC providers in the control municipality may be still exposed to (some of the) community actions implemented in the intervention municipality. For example, they may be exposed to a media appearance about the project. Again, exposure of PHC providers to the community actions will be closely measured in both the control group and in the intervention groups, which will allow us to detect and (if needed) control statistically for any contamination between study arms.

## CONCLUSION

This protocol describes the development and planned implementation of a package of community actions to support the delivery of brief alcohol advice in PHC in Colombia, Mexico and Peru. The protocol contributes to a better understanding of community actions which can be effective in supporting PHC providers to adopt brief alcohol advice in routine practice in Latin America, as well as in other regions. By improving our understanding of factors that can lead to increased delivery of brief alcohol advice in Latin America specifically, the community actions described in this protocol have the potential to support future efforts to scale-up PHC-based alcohol prevention activities in this region, leading to substantial public health benefits.
